# Investigating the Role of the Locus Coeruleus Noradrenergic System in Cognitive Function in Amnestic Mild Cognitive Impairment: An fMRI Study

**DOI:** 10.1192/j.eurpsy.2025.1697

**Published:** 2025-08-26

**Authors:** L. Penalba Sanchez, Y.-Y. Yi, E. Kurt, G. D. Femminella, C. Loane, M. Duckett, M. Callaghan, N. Weiskopf, R. Dolan, W. Glanz, M. Butryn, H. Mattern, M. Leiman, I. Mann, U. Pankratz, C. Y. Lübeck, N. Kelling, R. Howard, E. Düzel, D. Hämmerer

**Affiliations:** 1 Institute of Cognitive Neurology and Dementia Research, Otto Von Guericke University; 2 German Center for Neurodegenerative Diseases , Magdeburg, Germany; 3 Department of Neuroscience, Aziz Sancar Institute of Experimental Medicine, Istanbul University , Istanbul, Türkiye; 4 Department of Translational Medical Sciences, University of Naples Federico II, Naples, Italy; 5 Institute of Cognitive Neuroscience; 6 Wellcome Centre for Human Neuroimaging, University College London, London, United Kingdom; 7 Max Planck Institute for Human Cognitive and Brain Sciences, Leipzig, Germany; 8 Max Planck Centre for Computational Psychiatry and Ageing, London, United Kingdom; 9 Department Biomedical Magnetic Resonance; 10 Otto Von Guericke University, Magdeburg, Germany; 11: Institute of Psychiatry and the Wolfson Centre for Age Related Disease, Kings College, London, United Kingdom; 12 Center for Behavioral Brain Sciences, Magdeburg, Germany; 13 Department of Psychology, University of Innsbruck, Magdeburg, Austria

## Abstract

**Introduction:**

The Locus Coeruleus (LC), the first brain region affected by TAU aggregates in Alzheimer’s disease (AD), is the primary source of noradrenaline (NA). Given the importance of NA in cognitive functions, noradrenergic interventions may benefit patients with AD pathology.

**Objectives:**

This study aims (i) to examine memory delay and related fMRI activations in brainstem and midbrain regions in healthy aging and amnestic mild cognitive impairment (aMCI); and (ii) to explore the impact of atomoxetine on memory delay and inhibitory control in aMCI.

**Methods:**

For aim (i), event-related fMRI was used. Fifty-three subjects (28 healthy older adults and 25 with aMCI) completed an incidental recognition memory task with emotional and neutral images. Memory tests were administered four hours later, brain BOLD fMRI activations for remembered versus not remembered images were assessed. For aim (ii), seven participants attended the lab over four days. On visit 1, they received either a placebo or atomoxetine, followed by a stop signal task and an incidental memory task. On visit 2, they completed a recognition memory task. Visits 3 and 4 repeated this protocol. T-tests were used to compare results between groups and visits.

**Results:**

For aim (i), a greater activation in the left caudate nucleus was observed in older adults compared to aMCI when contrasting remembered items with not remembered ones (SVC, cluster-level pFWE-corr = 0.08). A significant increase in activation was also found in the locus coeruleus (SVC, cluster-level pFWE-corr = 0.018). However, after adjusting for LC integrity and global grey matter volume (GMV), these differences were no longer significant, suggesting structural changes contribute to LC activation differences between healthy controls and MCI participants. For aim (ii), inhibitory control improved slightly but was not statistically significant, while delayed memory decreased during the atomoxetine visit compared to the placebo visit (*p*<.05).

**Conclusions:**

Our findings highlight the caudate nucleus’s role in memory encoding in healthy older adults versus those with aMCI, linking LC dysfunction in aMCI to reduced LC integrity. The lack of improvement in executive functions and decreased memory during the atomoxetine visit may stem from individual differences in aMCI. Studies suggest atomoxetine is more effective in patients with high apathy and reduced LC integrity. In future analyses we will stratify participants by apathy and LC integrity to explore atomoxetine’s potential benefits. This study contributes to understanding neural mechanisms in aging and aMCI and informs personalized interventions for cognitive decline in AD.

**Disclosure of Interest:**

None Declared

